# Diffuse yellow-golden axillary plaques

**DOI:** 10.1016/j.jdcr.2024.12.032

**Published:** 2025-01-15

**Authors:** An Jian Leung, Aarav Singh Sandhu, Xin Yi Lee, Zhao Jian Oswald Lee, Adeline Mei Yen Yong

**Affiliations:** a Department of Dermatology, National University Hospital, Singapore; b Yong Loo Lin School of Medicine, National University, Singapore; c Department of Pathology, National University Hospital, Singapore

**Keywords:** xanthoma, myeloma, paraproteinemia, lipid

A 55-year-old Chinese female with iron deficiency anemia and chronic urticaria presented with a 1-year history of an asymptomatic yellowish discoloration over her axillae extending to her upper chest and inner upper arms. She was otherwise well and reported no new medications or contactants. Physical examination revealed thin yellow-golden plaques around bilateral axillae, upper chest, and inner arm with central axillary sparing (Figs 1, *A* and *B*). Liver and renal function tests, glycated hemoglobin, and lipid panel were normal. Her full blood count revealed mild microcytic anemia. A punch biopsy was performed (Fig 2) over the left axilla.

**Fig 1:**
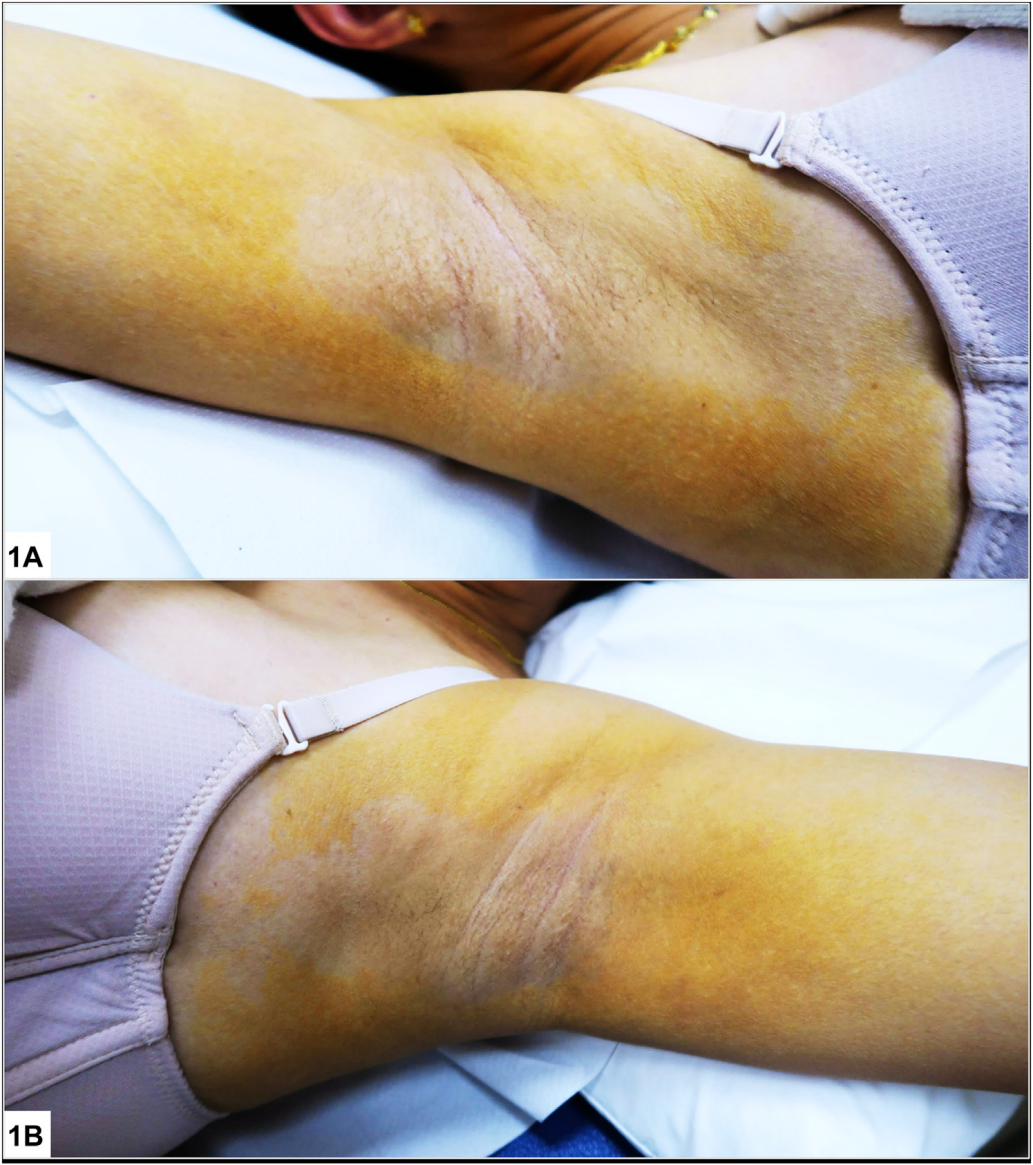

**Fig 2:**
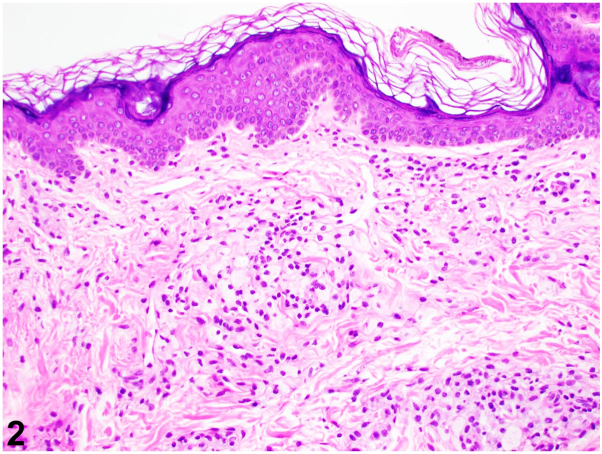

**Question 1: What is the most likely diagnosis?**

A. Contact dermatitis
B. Cutaneous amyloidosis
C. Diffuse plane xanthoma
D. Sarcoidosis
E. Systemic sclerosis

**Answers:**

A. Contact dermatitis – Incorrect. The lack of contactant history with the above histological findings makes this diagnosis extremely unlikely.
B. Cutaneous amyloidosis – Incorrect. While macular amyloidosis may affect the arm and axilla (like in this case), it would typically present as pruritic reticulated dark brown papules or plaques. Furthermore, focal eosinophilic faceted deposits in the papillary dermis with pigment incontinence would be seen on histology.
C. Diffuse plane xanthoma – Correct. Diffuse plane xanthoma (DPX) is a distinct form of non-Langerhans cell histiocytosis.^1^, ^2^, ^3^ Clinically, it manifests as yellow-golden to orange macules and plaques usually over the neck, axilla, folds, chest, and back. Dermoscopic examination reveals a yellow-orange amorphous superficial structure with occasional cholesterol crystals. An alabaster granule-like aggregate may be observed. Typical histological features include the presence of foamy CD68+ histiocytes, foam cells (as seen in this case), tortuan giant cells, and lymphocytic and eosinophilic dermal infiltrate.
D. Sarcoidosis – Incorrect. Skin manifestations in cutaneous sarcoidosis are erythema nodosum, discoid eczema, erythema multiforme, calcinosis cutis, or generalized pruritus. The lack of systemic symptoms and absence of noncaseating granulomas on histology make this diagnosis unlikely.
E. Systemic sclerosis – Incorrect. Cutaneous manifestations of systemic sclerosis include salt-pepper pigmentation, Raynaud's phenomenon, and sclerodactyly, among others.

**Question 2: Which of the following conditions are associated with the diagnosis?**

A. Diabetes mellitus
B. Ectopia lentis
C. Iron deficiency anemia
D. Liver impairment
E. Multiple myeloma

**Answers:**

A. Diabetes mellitus – Incorrect. While diabetes may be associated with hyperlipidemia in metabolic syndrome, which may put one at risk of developing cutaneous xanthomas, diffuse planar xanthoma is a distinct subtype of non-Langerhans histiocytosis unassociated with diabetes.
B. Ectopia lentis – Incorrect. This is associated with connective tissue diseases like Ehlers-Danlos, Marfan’s syndrome, and homocystinuria.
C. Iron deficiency anemia - Incorrect. While observed in our patient, this was an unassociated incidental finding.
D. Liver impairment – Incorrect. While DPX may rarely be associated with necrobiotic xanthogranuloma, which may have multiorgan involvement, it rarely affects the liver.^1^
E. Multiple myeloma – Correct. DPX is highly associated with hematological disorders like multiple myeloma, monoclonal gammopathy of undetermined significance, and lymphoproliferative disorders. It may precede hematological disease by years. Our patient was diagnosed with IgG-κ smoldering multiple myeloma. She declined a bone marrow aspirate and remains on close surveillance with a hematologist. Treatment involves addressing the underlying disease (where identified) and topicals or lasers if cosmetically bothersome.^4^^,^^5^

**Question 3: What lipid profile abnormality is most commonly observed?**

A. Elevated low-density lipoproteins
B. Elevated total cholesterol
C. Elevated triglycerides
D. Elevated very low-density lipoproteins
E. Normal lipid profile

Answers.

A. Elevated low-density lipoproteins – Incorrect. This may be associated with hyperlipidemia, familial hypercholesterolemia, and ischemic heart disease.^3^
B. Elevated total cholesterol – Incorrect. This is associated with hypercholesterolemia.
C. Elevated triglycerides – Incorrect. This is associated with eruptive xanthomas.
D. Elevated very low-density lipoproteins – Incorrect. This is associated with familial combined hypercholesterolemia, familial hypertriglyceridemia, and familial lipoprotein lipase deficiency.^3^
E. Normal lipid profile – Correct. A normal lipid profile, as observed in our patient, is typically seen.^1^ A proposed mechanism for cutaneous xanthomatosis is the binding of circulating pathogenic immunoglobulins with lipoproteins and cutaneous deposition of these immunoglobulin-lipoprotein complexes. Phagocytosis of these complexes by macrophages forms foam cells seen in histology.^1^, ^2^, ^3^

## Conflicts of interest

None disclosed.
